# The Analysis of Optometrist Primary Eye Care Professionals as a Specialized Practice across India's Different States

**DOI:** 10.4314/ejhs.v36i1.5

**Published:** 2026-01

**Authors:** Aleena Saifi, Sayema Afrin, Sachitanand Singh, Renu Thakur

**Affiliations:** 1 Chitkara School of Health Sciences, Chitkara University, Punjab, India; 2 Department of Optometry, UIAHS, Chandigarh University, Punjab, India; 3 Centre for Research Impact and Outcome, Chitkara University, Punjab, India

**Keywords:** Optometrist, Vision impairment, Specialty optometry, Contact lens, Binocular vision, Low vision

## Abstract

**Background:**

Optometrists play a pivotal role in preventing and managing vision impairment through the provision of comprehensive eye care services. In India, vision impairment remains a major public health challenge, largely driven by uncorrected refractive errors, cataract, and other avoidable causes. Despite recent regulatory advances, limited evidence exists on the scope and distribution of specialty optometry practices across Indian states. This study assessed the extent and composition of specialty optometry practices among optometrists across selected Indian states.

**Methods:**

A cross-sectional survey was conducted among practicing optometrists in six Indian states using a pre-validated, self-administered questionnaire distributed via Google Forms. Informed consent was obtained electronically. Data were analyzed using Microsoft Excel and SPSS version 20. Descriptive statistics were computed to summarize practice patterns.

**Results:**

A total of 325 responses were received, of which 321 were complete and included in the analysis. All participants reported providing refraction services, with a high monthly workload. Specialty practices were less common, with contact lens practice reported by 69.5%, binocular vision services by 62.0%, and low-vision care by 61.0% of respondents. The average number of specialty cases managed per month ranged from 5 to 15. Marked inter-state variations in specialty practice were observed, reflecting disparities in workforce distribution and service availability.

**Conclusion:**

The findings highlight a substantial reliance on refraction-based services and an insufficient level of specialty optometry practice relative to population needs. Strengthening specialty training and expanding the optometry workforce beyond routine eye examination are essential to reduce avoidable blindness in India.

## Introduction

Optometry is a self-regulated healthcare profession in many developed countries; however, in India, formal regulation was established only recently through the National Commission for Allied and Healthcare Professions Act, 2020 (1). Under the Indian Labour Code (2267), optometrists are recognized as primary eye care professionals providing comprehensive eye and vision care services across the lifespan.

Vision impairment and blindness remain significant public health concerns in India (2). Uncorrected refractive errors alone affect more than 11 million children, while the national optometrist-to-population ratio stands at approximately 1:219,000 (3). This workforce shortage underscores the need to understand current optometry practice patterns, particularly in specialty areas such as contact lenses, low-vision rehabilitation, pediatric optometry, and binocular vision.

Optometrists in India are required to hold a Bachelor's or Master's degree in Optometry, reflecting the profession's classification as a specialized healthcare discipline. Despite progress toward professional standardization and regulation, evidence on the real-world distribution and scope of specialty optometry services across Indian states remains limited (4). Variations in administrative systems, educational exposure, resource availability, and public awareness may influence practice patterns.

This study aims to examine the scope of specialty optometry practices across selected Indian states and to explore workforce-related challenges affecting the delivery of comprehensive eye care services.

## Methods

**Study design and setting**: A cross-sectional survey was conducted among practicing optometrists across six Indian states: Punjab, Haryana, Tamil Nadu, Kerala, Bihar, and Jharkhand.

**Participants**: Eligible participants were practicing optometrists holding at least a Bachelor's degree in Optometry. Opticians, interns, and vision care technicians were excluded.

**Sample size**: The sample size was calculated using Andrew Fisher's formula, assuming a 95% confidence level, a standard deviation of 0.5, a margin of error of 5.3%, and a population size of 49,000 optometrists. The minimum required sample size was 322.

**Data collection**: A pre-validated questionnaire (4) was administered online via Google Forms over a five-month period. The questionnaire included an electronic informed consent section. Snowball judgmental sampling was employed to recruit participants.

**Data analysis**: Data were analyzed using Microsoft Excel and SPSS version 20. Descriptive statistics (mean, minimum, and maximum values) were calculated. Participants were categorized by years of experience into early career (<1 year), early mid-career (1–3 years), and mid-career (>3 years).

**Ethical considerations**: The study adhered to the principles of the Declaration of Helsinki (1975, revised 2000).

## Results

Of the 325 survey responses received, 321 were complete and included in the analysis. Among participants, 46.4% were male and 53.6% were female. Approximately 30% had completed postgraduate education, including master's degrees and fellowships.

All respondents reported performing refraction services, with a mean monthly workload exceeding 600 cases. Specialty optometry practices were less frequently reported. Contact lens practice was reported by 69.5% of optometrists, followed by binocular vision (62.0%) and low-vision services (61.0%). The average number of specialty cases managed per month ranged from 5 to 15 in table 2.

**Table 2:** Descriptive statistics of optometry practice activities per month

Variable	Mean	Minimum	Maximum
Number of refractions performed per month	619.65	25	3000
Percentage of contact lens patients among total patients (%)	14.58	0	100
Number of contact lens fittings performed per month	15.44	0	150
Number of aftercare/follow-up examinations performed per month	8.77	0	90
Number of patients with convergence and accommodation anomalies per month	20.78	0	500

**Figure 1:**
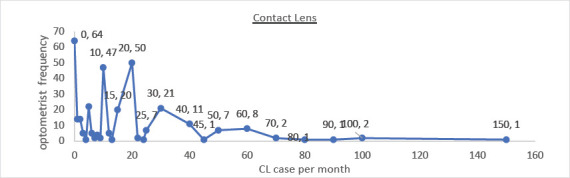
Monthly contact lens fits by an optometrist. It shows the frequency of optometrists doing the CL case in a month. The X-axis shows the number of CL cases seen per month; the y-axis represents the frequency of optometrists

**Figure 2:**
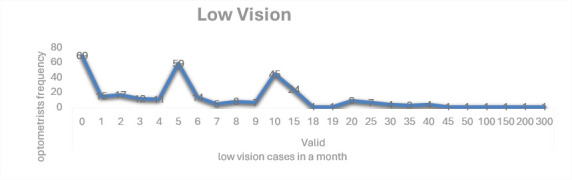
Low vision case per month. It shows the frequency of optometrists doing low vision cases in a month. The X-axis shows the number of low vision cases seen per month; the y-axis represents the frequency of optometrists

Contact lens services were primarily provided for keratoconus (30.5%), scleral lenses (24.0%), bi/multifocal lenses (16.4%), and orthokeratology (11.0%) figure 1. Low-vision services were provided by only 14% of respondents, with an average of 10 cases per month figure 2.

**Figure 3:**
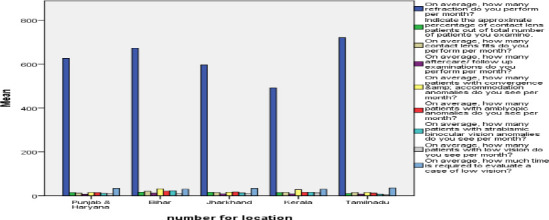
The mean values with respect to different states considered in this study. Refraction is done more by optometrists in Tamil Nadu. Convergence and accommodative anomalies and contact lens cases are mostly seen in Bihar. Low vision cases are detected higher in Kerala

Marked inter-state differences were observed. Optometrists in Tamil Nadu reported higher engagement in low-vision services, whereas contact lens services predominated in Punjab, Haryana, and Jharkhand as shown in figure 3. Female optometrists were more frequently involved in specialty practices. Early mid-career optometrists performed the highest volume of refractions, while mid-career practitioners managed more contact lens and low-vision cases.

The data was analysed in the mean, minimum, and maximum values of the questions are shown in Table 2. The mean value, maximum and minimum value of the data of each question is calculated with SPSS software. This gave us idea about the average case of each speciality done by an optometrist. It also shows the minimum and maximum cases seen per month. This study finds that refraction is done by all the optometrists in all five states of India. The average case of refraction done by an optometrist is 620. Maximum and minimum values are given in Table No. 2. Tamil Nadu is the state having the highest cases of refraction done by optometrists. The current study result shows that the specialty optometry practice rate is lower compared to the need for specialty practice in India. The Types of specialty practice in India, contact lenses percentage is 69.47%, binocular vision is 62%, and low vision is 61%. Contact lens is commonly practiced by optometrists, followed by binocular vision and low vision. The average of all specialty cases seen by an optometrist per month is 5-15 cases. The maximum and minimum cases seen in contact lens (Fig. 1), binocular vision, and low vision (Fig. 2) are shown in Tables No. 2 & 3. The current study result shows that the specialty contact lenses practiced by optometrists are as follow 30.50% are doing contact lens on only keratoconus patients, 24% of them are doing scleral contact lens, 16.40% doing bi/multifocal contact lens, and 11% are doing orthokeratology. The current study findings show that the average number of cases of low vision seen by an optometrist is 10 monthly as shown in Table No. 3. And only 14% of optometrists are seeing these cases. The finding for binocular cases, convergence, accommodative & amblyopic anomalies are commonly seen. The most common therapy given to binocular anomalies patients is Occlusion (85.50%), followed by Prism bar (47%), and Stereogram cards (45.30%). The least common is Synaptophore (35.90%).

**Table 3:** Descriptive statistics of specialty optometry cases and evaluation time

Variable	Mean	Minimum	Maximum
Number of patients with amblyopic anomalies per month	16.22	0	300
Number of patients with strabismic binocular vision anomalies per month	13.93	0	400
Number of patients with low vision per month	9.66	0	300
Time required to evaluate a low-vision case (minutes)	32.46	0	180

## Discussion

This study provides insight into the current scope of optometry practice in India and reveals a substantial imbalance between refraction services and specialty care. The high monthly refraction workload reflects both the burden of uncorrected refractive errors and the shortage of optometrists nationwide (3,5). Specialty services such as low-vision rehabilitation and binocular vision therapy remain underutilised despite significant population need.

Comparisons with international and regional studies suggest that barriers to specialty practice—including time constraints, limited training, lack of awareness, and cost—persist in the Indian context (6-8). Expanding access to advanced training, updating curricula, and promoting continuing professional development may help address these gaps.

Optometrists play a critical role in India's eye health system; however, current practice patterns are heavily skewed toward routine refraction. To effectively reduce avoidable blindness and visual impairment, India requires a larger and better-trained optometry workforce with competencies extending beyond routine eye examination. Strengthening specialty optometry practice should be prioritized within national eye health strategies.

A current study shows that optometrists perform one low vision case in an average time of 45 minutes which shows that its time consuming. The current study finds that optometrists are seeing 5-10 cases per month which is a very small number of cases seen by an optometrist as the prevalence of low vision is 1.03% (5). The prevalence of visual impairment in India is 2.55% of the overall population as per the NPCB report. And blindness prevalence is 0.36% (5). Low-vision practitioners are needed to provide services to visually impaired people. In India, more low-vision practitioners are required to handle these people. The present study shows that 69.4% of optometrists are contact lens practitioners which is similar to the result of a study done by Pradeep Paul George et al, 62% respondents are practicing contact lenses (9). In this study, the contact lens practitioners who practice contact lenses, among them only 2.5% are doing 50 cases per month, and 30.5% are practicing scleral contact lenses. There is a 2.3% prevalence of keratoconus in central India (10). And the prevalence of 28.9% of severe Dry eye in India (11). To handle keratoconus and severe dry eye in India requires more contact lens practitioners as keratoconus is majorly managed by RGP, & scleral contact lenses. As myopia progression is a huge burden in Asian countries the Ortho-K lenses are the choice for children to reduce myopia progression. The prevalence of myopia is 7.5% in 5-15 age group children and more in urban children as compared to rural (11). To reduce this burden contact lens practice should be encouraged. The current study finds that 62% of optometrists practice binocular vision among them amblyopic and strabismic anomalies are commonly seen. Nowadays, digital devices and near work have increased due to the demand of the work type which causes non-strabismic binocular vision anomalies and digital eye strain. In India, the prevalence of amblyopia is 1.75% (12), and the prevalence of strabismus is 0.7% in India (13), the prevalence of NSBVD was 31.5% in the urban population and 29.6% in the rural population (14), and the prevalence of digital eye strain was 45.5% in Indians (15). The prevalence of asthenopia symptoms in computer users is 87.5% in central India (16,17). The binocular assessment test will detect those anomalies that are performed by an optometrist and to treat these anomalies vision therapy is given, so to detect and treat these anomalies India needs more optometrists which are binocular vision specialists. The current study finds that occlusion is the most given therapy in amblyopia by optometrists and the prism bar therapy and stereogram card are given in NSBVD and strabismus whereas in the study of Patwardhan SD et al., the treatment offered is a pencil push-up test (79%) and synaptophore (18%) (18). Nowadays vision therapy has new technologies and treatments which should be used to treat all these anomalies other than older methods. The education and practice of new therapy are encouraged for optometrists (19).

In conclusion, this study has examined the vital and necessary function that optometrists perform in the delivery of primary eye care services. Their knowledge encompasses a broad range of crucial eye care services, going well beyond the prescription of corrective lenses. Thus, the present study concludes that India needs more qualified optometrist with expertise beyond Refraction and Routine Eye Examination to eliminate avoidable blindness. The study was limited by its sample size and the inclusion of selected states only. Future studies should include a larger, nationally representative sample.

## Figures and Tables

**Table 1:** Distribution of respondents by state in India (n = 321)

State	Frequency	Percent
Punjab & Haryana	80	24.9
Bihar	48	15.0
Jharkhand	59	18.4
Kerala	67	20.9
Tamil Nadu	67	20.9
Total	321	100.0
